# Knowledge of, and Attitudes Toward, Concussion in Japanese Male Collegiate Athletes

**DOI:** 10.3389/fspor.2022.835100

**Published:** 2022-02-18

**Authors:** Keita Suzuki, Takashi Imamoto, Satoshi Nagai, Masahiro Takemura

**Affiliations:** ^1^Sports Research and Development Core, University of Tsukuba, Ibaraki, Japan; ^2^Graduate School of Comprehensive Human Sciences, University of Tsukuba, Ibaraki, Japan; ^3^Faculty of Health Sciences, Tsukuba International University, Ibaraki, Japan; ^4^Faculty of Health and Sport Sciences, University of Tsukuba, Ibaraki, Japan

**Keywords:** recognizing symptoms, knowledge, reporting attitudes, reporting intention, collegiate student-athletes

## Abstract

Japan has no streamlined concussion education for collegiate athletes, and guidelines vary by sport. In particular, research on knowledge of, and attitudes toward, concussion is necessary for the establishment of concussion education for Japanese collegiate athletes. The aim of the present study was to assess the knowledge of, and attitudes toward, concussion in Japanese male collegiate athletes and to investigate their experiences with suspected concussion symptoms. An online questionnaire was administered to 390 collegiate athletes participating in the following five sports with a high incidence of concussion: rugby union, soccer, basketball, American football, and judo. Of the 121 valid responses, 91 (77.1%) indicated that they had experienced suspected concussion symptoms at least once and 46 of these 91 respondents had not reported their symptoms of suspected concussion at least once. Athletes who had never experienced concussion symptoms were significantly less likely to recognize the symptoms of concussion (*p* < 0.001). Most athletes recognized headache and dizziness as suspected concussion symptoms. However, the recognition rate for several symptoms was lower than the prevalence of these symptoms as shown in previous studies. This suggests that educational initiatives might be important to bridge the gap between athletes' knowledge and understanding of frequently occurring concussion symptoms and to improve reporting behavior.

## Introduction

Reporting the symptoms of suspected concussions depends on athletes' ability to recognize and attribute their symptoms, as many common symptoms are subjective (e.g., headache and dizziness). Several studies on knowledge of concussion have been conducted among collegiate athletes outside of Japan (Fedor and Gunstad, 2015; Beidler et al., 2018, 2021; Chapman et al., 2018; Bernstein et al., 2019; Anderson et al., 2021). These studies have investigated athletes' perceptions of symptoms based on the suspected concussion symptoms presented in the consensus statement (McCrory et al., 2017) and the Sports Concussion Assessment Tool 5 (SCAT 5) (Echemendia et al., 2017) while adding dummy symptoms. Many of them recognize the frequent symptoms, such as headaches (92.7–96.1%) and dizziness (89.3–90.3%) (Fedor and Gunstad, 2015; Beidler et al., 2018; Bernstein et al., 2019). Conversely, the recognition rate of emotional symptoms (e.g., being irritable, easily angered) is low (32.5–50.3%) (Fedor and Gunstad, 2015; Beidler et al., 2018; Bernstein et al., 2019). Many college athletes had experienced highly recognizable symptoms such as headaches (90.7–92.2%) and dizziness (68.9–69.5%), while fewer had experienced emotional symptoms such as irritability (13.2–15.2%) in the United States (Kerr et al., 2016a; Wasserman et al., 2016). Moreover, most of these previous studies were conducted with collegiate athletes in the United States. The degree of concussion awareness in the United States is considered high; secondary school athletes across the United States have received annual mandatory concussion education since 2014 (Gibson et al., 2015).

Athletes' attitudes and intentions toward reporting suspected concussion symptoms may also influence whether they report these symptoms when they become aware of them. Previous authors have investigated attitudes and intentions toward reporting concussion symptoms among college student-athletes (Kroshus et al., 2015; Chinn and Porter, 2016; Chapman et al., 2018; Bernstein et al., 2019) and found that collegiate student-athletes have a safety-conscious attitude toward reporting concussion symptoms; for example, they feel that having a concussion is a big deal and does not prove that they are tough (Bernstein et al., 2019). In addition, collegiate student-athletes with strong intentions to report concussion symptoms, as measured in the pre-season, are more likely to report those symptoms during the season (Kroshus et al., 2015). However, attitudes and intentions toward reporting concussion symptoms are likely influenced by individual personalities and national cultures. Given the unique Japanese attitude, behaviors, and culture, which include being modest in self-assertion, respecting others, and valuing harmony, we believe it is necessary to research this issue, especially with Japanese collegiate athletes.

Concussion education is one of the most important factors in ensuring that athletes are knowledgeable about concussions and have appropriate reporting attitudes. The US has a well-developed concussion education system. Beidler et al. (2021) classified concussion publicities into three degrees. Japan is probably at the moderate degree in the Beidler et al. (2021) classification. This means that Japan has no streamlined concussion education for collegiate athletes, and guidelines vary by sport. For example, in Japan rugby union, there are educational opportunities for coaches and medical staff, while athletes are not always guaranteed the opportunity to receive concussion education. Moreover, there have been no studies of concussion knowledge levels, reporting attitudes, or educational interventions among athletes in Japan. In order to design a concussion education system in Japan, it is necessary to clarify the knowledge that athletes have and their attitude toward concussion reporting.

Therefore, the aim of the present study was to assess the knowledge of, and attitudes toward, concussion in Japanese male collegiate athletes and to investigate their experiences with suspected concussion symptoms.

## Methods

### Participants

**Figure 1:**
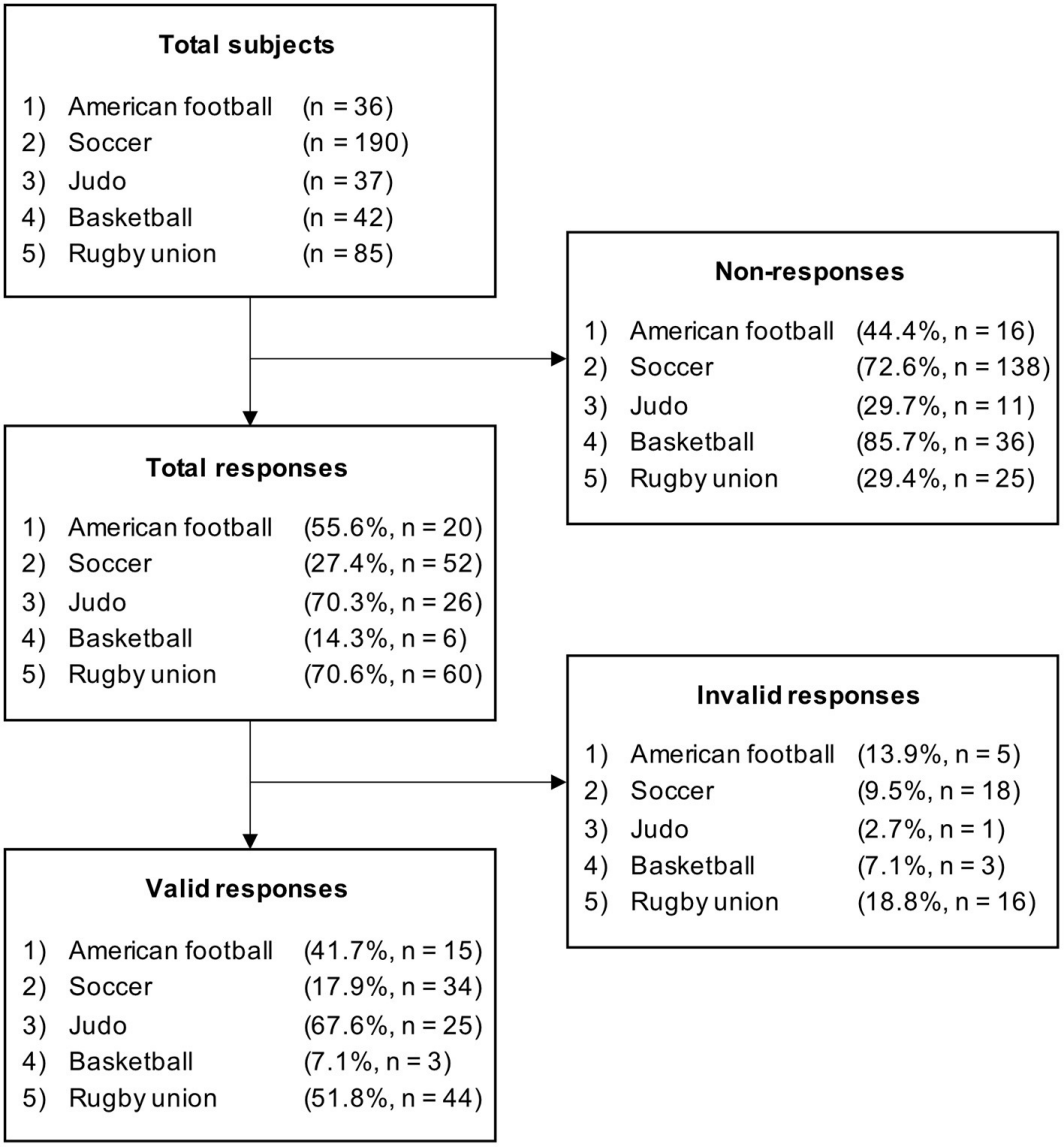
Valid response rate of participants in each sport.

This was a cross-sectional study of Japanese male collegiate student-athletes. We included five sports: American football, soccer, judo, basketball, and rugby union. These five target sports fulfilled the following two criteria: (1) a high reported incidence of concussions (Pfister et al., 2016; Akoto et al., 2018; Prien et al., 2018), and (2) a certain amount of physical contact. A total of 390 collegiate athletes participated in the study survey (Figure 1). The Ethics Committee of the Faculty of Health and Sport Science at the University of Tsukuba approved the study (Reference number: 020-104). All student-athletes provided written informed consent before participating.

### Measures

The questionnaire regarding knowledge and attitudes toward concussion was developed based on previous studies (Rosenbaum and Arnett, 2010; Kroshus et al., 2014; Kurowski et al., 2014; Kearney and See, 2017). It consisted of four sections: (1) demographics (age, academic year, sport, and years of sport), (2) experiences of suspected concussive events, (3) knowledge of concussion, and (4) attitudes toward reporting concussion. Student-athletes responded to an online questionnaire distributed using the SurveyMonkey tool (http://www.surveymonkey.com).

The experiences of suspected concussive events section comprised the following three questions:

1) Have you experienced any symptoms of suspected concussion?
2) If you have experienced symptoms of suspected concussion, have you reported those symptoms to anyone?
3) If you have experienced symptoms of suspected concussion, in which sports activity have you experienced those symptoms?

An experience of symptoms of suspected concussion was defined as “an athlete had experienced at least one of the symptoms of suspected concussion (Echemendia et al., 2017; McCrory et al., 2017) after a blow to the head, face, neck, or elsewhere on the body with an impulsive force transmitted to the head. Also, it did not matter whether they reported those symptoms to anyone or not, nor whether they were assessed by a doctor no not”.

In the concussion knowledge section, we calculated the Concussion Symptom Knowledge Index (CSKI) as the number of symptoms that an athlete recognized as suspected concussion symptoms out of 16 symptoms selected from the consensus statement (McCrory et al., 2017) and the Sports Concussion Assessment Tool 5 (Echemendia et al., 2017). A higher CSKI score indicated that an athlete had a better knowledge of suspected concussion symptoms. In addition, knowledge about concussion was assessed as such using a 13-item Concussion Knowledge Index (CKI), which consisted of true-false questions about the definition of concussion, symptoms of concussion, typical course recovery, Complications of concussion, and current recommendations on concussion management (Rosenbaum and Arnett, 2010; Kroshus et al., 2014; Kurowski et al., 2014; Kearney and See, 2017). We summed the number of correct answers to create a composite score, with higher scores on the CKI indicating a better knowledge of concussion.

The attitudes toward reporting concussion were measured with the following two scales: (1) Attitude toward reporting concussion (ARC) (Register-Mihalik et al., 2013a; Kroshus et al., 2014), and (2) intention to report symptoms (IR) (Tator, 2012; Chrisman et al., 2013; Kroshus et al., 2014). In the ARC, for example, participants responded to the question, “If I report what I suspect might be a concussion, my teammates will think less of me,” to what extent it would influence them to report concussion symptoms. In the IR, for example, participants answered whether they would report the symptom if they “had a headache” after an impact to the head. All items were scored on a 5-point Likert scale, with item scores summed. Higher ARC scores reflected the belief that there are more negative consequences of reporting a concussion; higher IR scores represented a greater intention to report symptoms. The internal consistency of each item in the ARC was low (Cronbach's α = 0.399); conversely, the internal consistency of each measurement in the IR was high (Cronbach's α = 0.809).

### Statistical Analysis

Questionnaires with answers to all required items were considered valid responses, but we excluded those where the answers were inconsistent (e.g., Respondents who simultaneously answered “I have experienced symptoms of suspected concussion” and "I have not experienced any symptoms).

We divided the participants into the following three groups based on their responses regarding their experience of suspected concussive events: (1) those who had never experienced any symptoms of suspected concussion (NE); (2) those who had experienced at least one symptom of suspected concussion and had not reported a symptom at least once (NR); and (3) those who had experienced at least one symptom of suspected concussion and in all cases had reported it themselves, or it had been detected by someone else (R).

The CSKI and CKI calculated the recognition rate and percentage of correct answers for each item, respectively. The chi-square test (Fisher's exact test with Monte Carlo estimates) was used to compare the differences in the recognition rates or the percentage of correct answers for each item between the three groups: NE, NR, and R. Following this line of analysis, the adjusted standardized residuals (ASR) from the contingency tables were analyzed to determine the differences between the three groups. The categories with ASR > 1.96 indicated that there were more cases than expected, whereas those with ASR < −1.96 indicate that there were fewer cases than expected (Field, 2013).

The CSKI, CKI, ARC, and IR scores were reported as means ± SD (standard deviations). The data were checked for normality using the Shapiro-Wilk normality test. Thereafter, the Kruskal-Wallis test (Fisher's exact test with Monte Carlo estimates) was used to compare the scores for CSKI, CKI, ARC, and IR between NE, NR, and R. As multiple comparisons were performed, a Bonferroni adjustment was applied to reduce the risk of Type I errors (*p* < 0.0167). All statistical analyses were performed using the SPSS version 26.0 package (IBM Japan Inc., Tokyo, Japan) with the significance level set at *p* < 0.05.

## Results

There were 121 valid responses (31.0%; Figure 1), of which basketball was excluded from the subsequent analysis because there were only three valid responses. The mean age was 20.2 ± 1.4 years, and the mean number of years athletes had been playing their current sport was 11.2 ± 4.9 years. Supplementary Table 1 shows the characteristics of the subjects with valid responses.

### Experiences of Suspected Concussive Events

Of the 118 respondents, 91 (77.1%) had experienced symptoms of suspected concussion at least once. Forty-six (50.5%) of these 92 respondents had not reported symptoms of suspected concussion at least once, that is, the NR group. Of the 91 participants, 47 experienced symptoms suggestive of concussion in matches (51.6%), 44 in practice sessions (48.4%).

### Knowledge of Concussion

The total CSKI score was significantly higher in the NR (9.3 ± 3.3) and R (9.2 ± 3.0) groups than in the NE group (5.8 ± 3.8) (*p* < 0.001; Table 1). The NE group had a significantly lower recognition rate for the following eight symptoms (*p* < 0.05): (1) headache (77.8%), (2) dizziness (59.3%), (3) confusion (37.0%), (4) nausea (63.0%), (5) vomiting (55.6%), (6) loss of consciousness (51.9%), (7) sensitivity to light or noise (3.7%), and (8) difficulty remembering (44.4%). Conversely, student-athletes in the NR group had a significantly higher recognition rate for the following three symptoms (*p* < 0.05): (1) headache (97.8%), (2) more emotional (17.4%), and (3) difficulty remembering (87.0%). In addition, the R group had a significantly higher rate of recognition of the following two symptoms (*p* < 0.05): (1) dizziness (93.3%), and (2) difficulty remembering (88.9%).

**Table 1:** Recognition rate of symptoms of suspected concussion.

	**Frequency of recognition**, ***N*** **(%)**				
**Concussion symptom knowledge index**	***R* (*N* = 45)**	**NR (*N* = 46)**	**NE (*N* = 27)**	**Total (*N* = 118)**	*p* ^*^
Headache	41 (91.1)	45 (97.8)^#^	21 (77.8)^§^	107 (90.7)	0.014
Dizziness	42 (93.3)^#^	39 (84.8)	16 (59.3)^§^	97 (82.2)	0.001
Nausea	39 (86.7)	40 (87.0)	17 (63.0)^§^	96 (81.4)	0.020
Difficulty remembering	40 (88.9)^#^	40 (87.0)^#^	12 (44.4)^§^	92 (78.0)	<0.001
Loss of consciousness	39 (86.7)	38 (82.6)	14 (51.9)^§^	91 (77.1)	0.002
Balance problems	38 (84.4)	34 (73.9)	16 (59.3)	88 (74.6)	0.059
Vomiting	35 (77.8)	37 (80.4)	15 (55.6)^§^	87 (73.7)	0.048
Blurred vision	29 (64.4)	28 (60.9)	12 (44.4)	69 (58.5)	0.231
Confusion	31 (68.9)	27 (58.7)	10 (37.0)^§^	68 (57.6)	0.031
Difficulty concentrating	19 (42.2)	21 (45.7)	6 (22.2)	46 (39.0)	0.125
“Pressure in head”	17 (37.8)	15 (32.6)	4 (14.8)	36 (30.5)	0.113
Fatigue or low energy	15 (33.3)	15 (32.6)	3 (11.1)	33 (28.0)	0.084
Drowsiness	11 (24.4)	16 (34.8)	4 (14.8)	31 (26.3)	0.163
Feeling like “in a fog”	6 (13.3)	13 (28.3)	5 (18.5)	24 (20.3)	0.202
Sensitivity to light or noise	10 (22.2)	12 (26.1)	1 (3.7)	23 (19.5)	0.057
More emotional	3 (6.7)	8 (17.4)^#^	0 (0.0)	11 (9.1)	0.038
Total CSKI score, mean (SD)	9.2 (3.0)^¶^	9.3 (3.3)^¶^	5.8 (3.8)	8.4 (3.6)	<0.001^†^

*R, Reported symptoms; NR, Non-reported any symptoms; NE, No experience of symptoms; CSKI, Concussion Symptom Knowledge Index; SD, standard deviation.*

* *P-value based on chi-square test (Fisher's exact test using Monte Carlo estimates).*

# *Indicating adjusted standardized residual was >1.96 (p < 0.05).*

§ *Indicating adjusted standardized residual was lower than −1.96 (p < 0.05).*

† *P-value based on the Kruskal-Wallis test (Fisher's exact test using Monte Carlo estimates).*

¶ *The score was significantly higher than that of NE (p < 0.001)*.

The total CKI score was higher in the NR (10.4 ± 1.4) and R (9.7 ± 1.3) groups than in the NE group (9.4 ± 1.5), although this was not significant (*p* = 0.067; Table 2). The NE group had a significantly lower frequency of answering the following question correctly: a concussion can only occur if there is a direct hit to the head (70.4%). Conversely, student-athletes in the NR group had a significantly higher percentage of correct answers to the question above (93.5%) (*p* = 0.030; Table 2).

**Table 2:** Percentage of correct answers for knowledge of concussion.

	**Frequency of Answering Correctly**, ***N*** **(%)**				
**Concussion knowledge Item (True or False)**	**R (*N* = 45)**	**NR (*N* = 46)**	**NE (*N* = 27)**	**Total (*N* = 121)**	*p* ^*^
In order to be diagnosed with a concussion, you have to be knocked out. (False)	45 (100.0)	44 (95.7)	25 (92.6)	114 (96.6)	0.219
An athlete needs to follow the process of recovery and then return to sport participation after a concussion according to a graduated stepwise rehabilitation strategy. (True)	42 (93.3)	46 (100.0)	24 (88.9)	112 (94.9)	0.100
People who have had one concussion are more likely to have another concussion. (True)	39 (86.7)	44 (95.7)	25 (92.6)	108 (91.5)	0.277
There is rarely a risk to long-term health and wellbeing from multiple concussions. (False)	43 (95.6)	42 (91.3)	23 (85.2)	108 (91.5)	0.341
Sometimes a second concussion can help a person remember things that were forgotten after the first concussion. (False)	41 (91.1)	42 (91.3)	23 (85.2)	106 (89.8)	0.736
Resting your brain by avoiding things such as playing video games, texting, and doing schoolwork is important for concussion recovery. (True)	37 (82.2)	42 (91.3)	24 (88.9)	103 (87.3)	0.409
If you receive one concussion and you have never had a concussion before, you will become less intelligent. (False)	38 (84.4)	41 (89.1)	22 (81.5)	101 (85.6)	0.632
A concussion can only occur if there is a direct hit to the head. (False)	38 (84.4)	43 (93.5)^#^	19 (70.4)^§^	100 (84.4)	0.030
An athlete who gets knocked out after getting a concussion is experiencing a coma. (False)	26 (57.8)	32 (69.6)	18 (66.7)	76 (64.4)	0.504
Being knocked unconscious always causes permanent damage to the brain. (False)	33 (73.3)	24 (52.2)	18 (66.7)	75 (63.6)	0.109
After a concussion occurs, brain imaging (e.g., CAT Scan, MRI, X-Ray, etc.) typically shows visible physical damage (e.g., bruise, blood clot) to the brain. (False)	19 (42.2)	20 (43.5)	17 (63.0)	56 (47.5)	0.188
After 10 days, symptoms of a concussion are usually completely gone. (True)	18 (40.0)	21 (45.7)	10 (37.0)	49 (41.5)	0.788
Younger people have a higher chance of concussion. (True)	14 (31.1)	22 (47.8)	7 (25.9)	43 (36.4)	0.119
Total CKI score, mean (SD)	9.7 (1.3)	10.4 (1.4)	9.4 (1.5)	9.8 (1.4)	0.067^†^

*R, Reported symptoms; NR, Non-reported any symptoms; NE, No experience of symptoms; CKI, Concussion Knowledge Index; SD, standard deviation.*

* *P-value based on chi-square test (Fisher's exact test using Monte Carlo estimates).*

# *Indicating adjusted standardized residual was >1.96 (p < 0.05).*

§ *Indicating adjusted standardized residual was lower than −1.96 (p < 0.05).*

† *P-value based on the Kruskal-Wallis test (Fisher's exact test using Monte Carlo estimates)*.

### Attitudes Toward Reporting Concussion

There were no statistically significant differences in the total ARC score and each item's score between the three groups (Supplementary Table 2).

The total IR score was higher in the NR group than in the NE and R groups, although the difference was not significant (*p* = 0.190; Table 3). Student-athletes in the NE group had significantly greater intentions to report a symptom of “seeing stars” after an impact to the head (*p* = 0.022). Also, student-athletes in the R group had significantly greater intentions to report two following symptoms: (1) “seeing stars” after an impact to the head (*p* = 0.022); (2) “having problems concentrating on the task at hand” after an impact to the head (*p* = 0.048).

**Table 3:** Intention to report symptoms.

	**Mean (SD)**				
	**R (*N* = 45)**	**NR (*N* = 46)**	**NE (*N* = 27)**	**Total (*N* = 121)**	*p* ^*^
See stars	3.7 (1.1)^#^	3.2 (1.2)	3.8 (1.0)^#^	3.5 (1.1)	0.022
Vomit or feel nauseous	4.2 (0.9)	4.1 (1.0)	4.4 (0.7)	4.2 (0.9)	0.326
Have a hard time remembering things	4.2 (0.8)	4.0 (0.8)	4.2 (0.9)	4.1 (0.9)	0.392
Have problems concentrating on the task at hand	3.7 (1.0)^#^	3.1 (1.0)	3.4 (1.0)	3.4 (1.0)	0.048
Feel sensitive to light or noise	3.6 (1.1)	3.4 (1.0)	3.3 (1.0)	3.4 (1.1)	0.340
Have a headache	3.8 (1.0)	3.5 (0.9)	3.9 (1.1)	3.7 (1.0)	0.110
Experience dizziness or balance problems	4.3 (0.6)	4.3 (0.7)	4.3 (0.8)	4.3 (0.7)	0.991
Feel sleepy or in a fog	4.0 (0.8)	4.0 (0.8)	3.9 (1.2)	4.0 (0.9)	0.991
Total IR score	31.4 (5.0)	29.5 (4.4)	31.1 (5.8)	30.6 (4.9)	0.190

*R, Reported symptoms; NR, Non-reported any symptoms; NE, No experience of symptoms; IR, Intention to report symptoms; SD, standard deviation.*

*Score 1 (Strongly disagree); Score 3 (Neutral); Score 5 (Strongly agree). The higher the score, the more desirable and safer the thinking and attitude about reporting a concussion.*

* *P-value based on Kruskal-Wallis test (Fisher's exact test using Monte Carlo estimates).*

# *The score was significantly higher than that of NR (p < 0.0167)*.

## Discussion

This study investigated the knowledge of and attitudes toward concussion in Japanese collegiate student-athletes. The results demonstrated that student-athletes who had had no experience of any symptoms had significantly lower recognition rates of the symptoms of a suspected concussion. Conversely, student-athletes who had failed to report a symptom at least once were more negative about reporting concussion, although this was not statistically significant.

### Experiences of Suspected Concussive Events

A total of 77.1% of the Japanese student-athletes reported experiencing suspected concussion symptoms, which is higher than in previous studies (Kurowski et al., 2014; Kerr et al., 2016b). Previous studies covered more sports than the present study, including sports with less physical contact (e.g., baseball and fencing); between 23.4 and 26.9% of all athletes experienced symptoms of suspected concussion (Kurowski et al., 2014; Kerr et al., 2016b). Conversely, the present study included only five sports with a large athletic population and a high incidence of concussion. We believe that this is the reason why the proportion of athletes who experienced symptoms of suspected concussion was higher than in previous studies.

Half of the athletes who had experienced suspected concussion symptoms did not report these symptoms; this result was in line with previous studies (Chinn and Porter, 2016; Kerr et al., 2016b; Wallace et al., 2017; Baugh et al., 2019). As the present study was a retrospective study, it is unclear how many of the unreported cases would have been removed from the activity if a doctor had assessed them. In unreported cases, where concussed athletes are not properly managed, there is a potential risk of sustaining a serious brain injury from an impact to the head with residual concussion symptoms. Thus, it is important to reduce the number of unreported cases to protect athletes during their long careers.

### Knowledge of Concussion

Over 80.0% of the Japanese athletes recognized headache, dizziness, and nausea as symptoms of a suspected concussion; this result concurred with those of previous studies (Register-Mihalik et al., 2013b; Cournoyer and Tripp, 2014; Fedor and Gunstad, 2015; Beidler et al., 2018; Bernstein et al., 2019). However, the recognition rate of six other symptoms, such as “more emotional” and “sensitivity to light or noise”, was <30.0%. These symptoms had lower recognition rates than previous studies among collegiate athletes (Fedor and Gunstad, 2015; Beidler et al., 2018; Bernstein et al., 2019). In previous studies, sensitivity to light or noise symptoms also occurred more frequently in responses (48.9–50.7% and 31.5–31.7%, respectively) than the recognition rate in the present study (Kerr et al., 2016a; Wasserman et al., 2016). Thus, in our cohort, it is likely that athletes would not report any unusual sensations, such as sensitivity to light or noise, if they did not recognize them as concussion symptoms. This suggests that it is important to bridge the gap between athletes' knowledge and perceptions of frequent concussion symptoms to improve reporting behavior.

Athletes who had never experienced concussion symptoms were significantly less likely to recognize them than those who had experienced symptoms. Chinn and Porter (2016) explored whether a history of concussion and exposure to concussion education were related to concussion knowledge. In this study, a history of concussion was not related to concussion knowledge, while concussion education was associated with increased concussion knowledge scores (Chinn and Porter, 2016). Our study did not include a survey on exposure to concussion education, therefore, we do not know whether the lack of recognition of concussion symptoms is attributable to a lack of exposure to concussion education. Future research would need to investigate the correlation between exposure to concussion education and concussion knowledge.

Athletes who had had no exposure to concussion symptoms gave significantly more incorrect answers to the following question: “a concussion can only occur if there is a direct hit to the head”. This result suggests that some athletes might think that it is not a concussion if there was no direct blow to the head; even if they recognize the concussion symptoms. Thus, it is important to educate athletes properly on how concussions can occur.

### Attitudes Toward Reporting Concussion

Regardless of the history of concussion and symptom reporting, athletes felt negative about reporting concussion symptoms because they would not be allowed to start playing or practicing when they thought they were ready; the mean Likert-score here was 4.1/5.0 (82.0%). Several previous studies have shown that the common reason for non-disclosure of concussion symptoms was that “they did not want to leave the game/practice (Kerr et al., 2016b; Beidler et al., 2018, 2021)”. In other words, continuing in a match or practice is a high priority for athletes. In addition, many athletes did not report concussion symptoms because they did not know it was a concussion and/or did not think it was serious enough (Kerr et al., 2016b; Beidler et al., 2018, 2021). These findings suggest that education about signs and symptoms might not be sufficient on its own to improve reporting behavior. We believe concussion education is necessary for improving athletes' attitudes toward concussion in order to address the dangers of continuing to play with concussion symptoms, including the risk of catastrophic injury or increased playing time lost.

Athletes who reported concussion symptoms were significantly more positive about reporting when they had problems concentrating on the task at hand; however, the recognition rate of this symptom was <50.0% in our cohort (Table 1). In this study, the questionnaire section on concussion knowledge was located before the section on reporting intentions, which may have influenced the responses about reporting intentions. Thus, the intention to report may have been more positive, even for symptoms with low recognition rates. However, Rawlins et al. (2020) reported that a one-point increase in knowledge score predicted a significant increase in symptom reporting intentions. Japanese athletes are not mandated to have the opportunity to be educated about concussions. In Japan, coaches and medical staff are provided with opportunities to educate themselves about concussions. However, if the staff does not provide opportunities for players to receive information about concussions, the players themselves will have to act to obtain the information themselves. Therefore, education about the concussion symptoms may contribute to improved reporting intentions as well as athletes' perception of symptoms.

While it is clear that concussion education is essential, it is also true that education does not directly lead to behavior change (Malcolm et al., 2021). Therefore, the focus should also be on the environment surrounding the athletes in Japan. Travis et al. (2021) pointed out that in British American football, medical support for players is often insufficient, influencing the diagnosis and management of players' return from injury. This situation is probably the same in amateur and youth sports in Japan. Sufficient medical support would make it easier for people to seek help when experiencing symptoms that cannot be detected or detected by concussion.

## Conclusions

This study is the first to investigate the experiences, knowledge, and attitudes of Japanese collegiate athletes' to suspected concussion symptoms. In the five sports studied that historically report a high incidence of concussion, 91 of the 118 respondents (77.1%), had experienced symptoms of suspected concussion at least once. Forty-six (50.5%) of the 91 respondents did not report suspected concussion symptoms at least once. Most collegiate athletes recognized headache and dizziness as symptoms of suspected concussion, which is similar to previous studies, however the recognition rate was lower for several other symptoms associated with concussion. This suggests that it is likely important to bridge the knowledge gap between athletes' perceptions and frequent concussion symptoms in order to improve reporting behavior and manage athletes in Japan more effectively. It is important to improve the concussion education system in Japan and simultaneously focus on improving medical support for amateur and youth athletes.

## Data Availability Statement

The raw data supporting the conclusions of this article will be made available by the authors, without undue reservation.

## Ethics Statement

The studies involving human participants were reviewed and approved by the Ethics Committee of the Faculty of Health and Sport Science at the University of Tsukuba. The ethics committee waived the requirement of written informed consent for participation.

## Author Contributions

KS, TI, SN, and MT: study conception and design. TI: questionnaire implementation. KS and TI: analysis and interpretation of the data. KS: paper composition. SN and MT: writing advice. All authors approved the final version of the manuscript.

## Funding

Financial support was provided by grants-in-Aid for scientific research from the Japan Society for the Promotion of Science KAKENHI (Grant Number 19K20009).

## Conflict of Interest

The authors declare that the research was conducted in the absence of any commercial or financial relationships that could be construed as a potential conflict of interest.

## Publisher's Note

All claims expressed in this article are solely those of the authors and do not necessarily represent those of their affiliated organizations, or those of the publisher, the editors and the reviewers. Any product that may be evaluated in this article, or claim that may be made by its manufacturer, is not guaranteed or endorsed by the publisher.
