# Supplementary material for: Knowledge of, and Attitudes Toward, Concussion in Japanese Male Collegiate Athletes
Source: Front Sports Act Living. 2022 Feb 18;4:835100. doi: 10.3389/fspor.2022.835100 (PMC8894241; doi:10.3389/fspor.2022.835100)
Supplement: Supplementary file 1 [file Data_Sheet_1.docx]

Supplemental table 1 Participant Demographics (N = 118)

|  | N | (%) ^*^ |
| --- | --- | --- |
| Sport |  |  |
| American football | 14 | (11.9) |
| Soccer | 34 | (28.8) |
| Judo | 25 | (21.2) |
| Rugby union | 45 | (38.1) |
| Year in college |  |  |
| Freshman | 47 | (39.8) |
| Sophomore | 24 | (20.3) |
| Junior | 29 | (24.6) |
| Senior | 18 | (15.3) |
| Experience of symptoms of suspected concussion |  |  |
| Yes | 91 | (77.1) |
| No | 27 | (22.9) |
| Did you report any symptoms of suspected concussion? |  |  |
| Did not report any symptoms | 46 | (39.0) |
| Reported or someone else detected symptoms | 45 | (38.1) |
| No experience of symptoms | 27 | (22.9) |

*^*^ Percentage of total sample*

Supplemental table 2 Attitude toward Reporting Concussion

|  | Mean (SD) | | | | | | | |  | |
| --- | --- | --- | --- | --- | --- | --- | --- | --- | --- | --- |
|  | R | (N = 45) | NR | (N = 46) | NE | (N = 27) | Total | (N = 121) | | p^#^ |
| If I report what I suspect might be a concussion, I will hurt my team’s performance. | 2.3 | (1.2) | 2.8 | (1.2) | 2.7 | (1.4) | 2.6 | (1.3) | | 0.085 |
| If I report what I suspect might be a concussion, I will not be allowed to start playing or practicing when I think I’m ready. | 4.0 | (0.7) | 4.2 | (0.7) | 4.1 | (0.7) | 4.1 | (0.7) | | 0.491 |
| If I report what I suspect might be a concussion, I will lose my spot in the lineup. | 1.8 | (1.0) | 2.0 | (1.0) | 2.0 | (0.9) | 1.9 | (1.0) | | 0.428 |
| If I report what I suspect might be a concussion, my teammates will think less of me. | 1.8 | (0.9) | 1.7 | (0.8) | 1.8 | (1.0) | 1.8 | (0.9) | | 0.985 |
| The sooner I report a concussion, the sooner I’ll be back at full strength.^*^ | 2.4 | (1.1) | 2.5 | (1.1) | 2.3 | (1.0) | 2.4 | (1.1) | | 0.568 |
| If I report what I suspect might be a concussion, I will be held out of upcoming games even if it is not a concussion. | 3.0 | (0.9) | 2.9 | (1.1) | 3.4 | (1.3) | 3.0 | (1.1) | | 0.237 |
| If I report what I suspect might be a concussion, my teammates will think I made the right decision.^*^ | 2.1 | (0.8) | 2.1 | (0.7) | 1.9 | (0.7) | 2.1 | (0.7) | | 0.436 |
| If I report what I suspect might be a concussion, I will be better off in the long run.^*^ | 3.2 | (1.0) | 3.6 | (0.8) | 3.2 | (1.0) | 3.4 | (0.9) | | 0.101 |
| Total ARC score | 20.6 | (3.9) | 21.9 | (3.1) | 21.4 | (3.6) | 21.3 | (3.6) | | 0.297 |

*Abbreviations. R, Reported symptoms; NR, Non-reported any symptoms; NE, No experience of symptoms; ARC, Attitude toward reporting concussion; SD, standard deviation.*

*Note. Score 1 (Strongly disagree); Score 3 (Neutral); Score 5 (Strongly agree). The higher the score, the more negatively they think about reporting a concussion.*

*^*^ Item has been reverse-coded. The lower the score, the more negatively they think about reporting a concussion.*

*^#^ P value based on Kruskal-Wallis test (Fisher's exact test using Monte Carlo estimates).*
